# Tiredness, depression, and sleep disorders in frontline healthcare workers during COVID-19 pandemic in Vietnam: A field hospital study

**DOI:** 10.3389/fpsyt.2022.984658

**Published:** 2022-10-17

**Authors:** Sy Duong-Quy, Si Tran-Duc, Dinh Hoang-Chau-Bao, Khue Bui-Diem, Quan Vu-Tran-Thien, Vinh Nguyen-Nhu

**Affiliations:** ^1^Sleep Lab Center, Lam Dong Medical College and Bio-Medical Research Center, Dalat, Vietnam; ^2^Sleep Lab Unit, Outpatient Department, Pham Ngoc Thach Medical University, Ho Chi Minh City, Vietnam; ^3^Immuno-Allergology Division, Hershey Medical Center, Penn State Medical College, Hershey, PA, United States; ^4^Department of Respiratory Functional Exploration, University Medical Center, University of Medicine and Pharmacy, Ho Chi Minh City, Vietnam; ^5^Department of Physiology–Pathophysiology–Immunology, University of Medicine and Pharmacy, Ho Chi Minh City, Vietnam

**Keywords:** COVID-19, sleep disorders, depression, burnout, frontline health workers

## Abstract

**Background:**

The COVID-19 outbreak witnessed in the autumn of 2021 led to unprecedented changes in healthcare systems in some emerging countries. Many field-hospitals, temporary sites of care for COVID-19 patients, were built around the country and followed by the healthcare workers who were mobilized. This study aimed to measure sleep disorders, depression, and fatigue in volunteers working at field hospitals during the COVID-19 outbreak.

**Methods:**

This was a cross-sectional study. The self-report questionnaire was used for each study subject. Sleep characters, including STOP's elements were questioned. Healthcare workers' burnout was detected by using Pichot's questionnaire.

**Results:**

One hundred front-line healthcare workers (FHWs), predominantly last year and graduated medical students, were included in the study (86% female subjects). The mean sleep-time of FHWs before, while working, and during the isolation period after working at COVID-19 field hospitals were: 7.78 ± 1.48, 5.71 ± 1.40, and 8.78 ± 2.31 h per day, respectively. Burnout was not a crucial issue for these volunteer subjects. The mean scores of Pichot's Fatigue Scale and Pichot's Depression Scale, measured after 4 weeks working at field hospitals, were 4.18 ± 5.42 and 2.54 ± 3.36, respectively. Thirteen participants were suspected of depression. The fatigue scores decreased significantly in the group who claimed short sleep latency. The factor that increased the depression score was “anxious feeling” (*p* = 0.001). Other significant factors were “short sleep latency,” “observed sleep apnea,” “tiredness, daily sleepiness” and “snoring.”

**Conclusion:**

Appropriate work schedule, better sleep conditions, and mental health support could be helpful for FHWs. The mandatory 2 weeks of isolation after working in field hospitals provided opportunity for FHWs' recovery.

## Introduction

The COVID-19 pandemic is the deadliest since the 1918 Spanish influenza and is still an ongoing challenge. From the beginning of the COVID-19 pandemic to June 8th, 2022, as stated by WHO, 530,896,347 confirmed cases and 6,301,020 cumulative deaths have been reported worldwide (1). In Vietnam, even after several months of implementation of the Zero-COVID strategy, the virus has spread rapidly since April 2021. Currently, more than 10,727,918 people have been infected and 43,081 people died (2). Despite being affected much later than other countries, the healthcare system in Vietnam was also boosted. Volunteer healthcare workers around the country were mobilized to help the most affected areas. In this context, healthcare providers and medical students from the provinces or cities with low rates of COVID-19 cases, voluntarily moved to those areas with high levels of COVID-19 to work at the COVID-19 field hospitals.

Physician fatigue, also known as burnout, is a highly prevalent but often underrecognized result of workplace stressors (3, 4). The consequences of burnout can include poor work-life integration, isolation, depression, and suicide. The study on the intern doctors in China showed that burnout may have serious implications not only for the quality of emotion and their professional efficacy but also for their health and wellbeing (5). Given the context COVID-19 front-line healthcare workers (FHWs: people working in all healthcare sectors such as physicians, assistant physicians, nurses, pharmacists, medical technicians, graduated, or last year medical students) had to face, under constant high risk of infection; it makes sense that they suffered from stress, work overload, and lack of sleep (6). Reciprocally, sleep disorders and burnout have been shown to be associated with risk of COVID-19 infection (7). Since the beginning of the pandemic, Kang and colleagues also reported that up to 34.4% of medical staff working in Wuhan suffered from mild mental health disorders and emphasized the need for mental health care for FHWs fighting the pandemic (8). Approximately 30% of the internists and primary care physicians who participated in a Japanese study had symptoms of burnout, anxiety, and insomnia, whereas 15% were depressed during the COVID-19 pandemic (9).

The present study aimed to demonstrate the psychological struggles of FHWs, which focused on tiredness, depression, and sleep disorders in those caring for COVID-19 patients. This study also analyzed the main factors related to the psychological struggles of the FHWs and the efficacy of a 14-day self-isolation period to help recover from any psychological stressors burdened during a FHW's time fighting COVID-19.

## Methods

### Study design and participants

This multi-center, prospective observational study looked at different groups of volunteer healthcare workers of Lam Dong province who came to Binh Duong province and Ho Chi Minh City–Vietnam. These volunteer healthcare workers were physicians, pharmacists, nurses, medical technicians, and graduated or last year medical students. They came from healthcare centers and Lam Dong Medical College to be mobilized to take care of patients with COVID-19 in the field hospitals of other cities (called FHWs).

Participants were free to fill the questionnaire on Google Form, developed by the Scientific Committee of Vietnamese Society of Sleep Medicine (VSSM), during their working and isolation periods. The isolation period was the obligated two-week time for all FHWs after they finished working in the field-hospitals and came back to Lam Dong province. This isolation period was reserved only for FHWs without COVID-19 infection after their mission. FHWs with COVID-19 infection during their mission in the field-hospitals were excluded from the present study.

The content of these questionnaires was also published on the local society website (https://forms.gle/oR9aaWLtE6oELVK67). The present study ensured confidentiality and provided an explanation without inducement for any unclear questionnaire items. Data collection closed at the end of the 2 weeks of isolation to describe sleep disorders and exhaustion during their frontline mission and recovery time. There were also no formal hypotheses being implemented to drive the sample size and calculation and all persons were included in the study. The study was approved by Institutional Review Board (IRB) of Lam Dong Medical College (no. 07.2021/NCKH-LMC).

### Questionnaire

Because the SARS-CoV-2 pandemic work in the field hospitals was unprecedented, the working period at the field hospitals was relatively short. Thus, an available simple questionnaire, which included 4 items of STOP score, was used in the present study instead of other complicated sleep scales previously used to diagnose the risk of sleep breathing disorders such as obstructive sleep apnea (OSA). It included four yes/no questions related to snoring, tiredness during the daytime, observed apnea during sleep, and hypertension.

FHW's tiredness was measured using the Pichot's Fatigue scale. This is a practical scale, consisting of 8 questions (items), scored progressively from “0” (not at all) to “4” (extremely). The score ranged from 0 to 32 and a total score above 22 revealed excessive fatigue. Additionally, the Pichot's Depression scale, measuring depression, consists of 13 binary questions and yields a score of 0 to 13. A total score above 7 indicated a depressive mood. The Pichot scale allows us to determine the possible role played by depression on possible cognitive impairment (10).

The questionnaire also includes socio-demographic data, medical problems such as cardiovascular problems or pulmonary disorders, depression, sleep habits, television and smartphone use, and night shift work.

### Data analysis

All the questionnaires received were checked by double blind verification to assure the data's validity and reliability. The present study expressed descriptive data as mean (SD) or median (IQR) for continuous variables and number/percentage (%) for categorical variables. This study assessed differences between “before the frontline working period,” “during the frontline working period,” and “during the post-working isolation period” using the two-sample *t*-test for continuous variables and Chi square for categorical variables. Tests were two-sided with significance set at < 0.05. The Stata 13.0 software was applied for all analyses.

## Results

### General characteristics of study participants

One hundred FHWs from Lam Dong province were included in the present study. Most of these FHWs in COVID-19 field hospitals were female medical students and still young (Table 1). More than half of the participants (*n* = 58) claimed to be physically active. The mean active time was 2.30 h/day for the total study sample. There was only one participant with hypertension and three others with depressive states.

**Table 1:** General characteristics of study participant (*n* = 100).

**Characteristics**		**%**
Gender	Female	86.0
	Male	11.0
	Others	3.0
Education level	College	79.0
	University	13.0
	Post-graduated	8.0
Career	Physicians and nurses	6.0
	Graduated students*	28.0
	Last year students**	66.0
Marital situation	Single	90.0
	Married	10.0
Age class	From 18 to 25 years old	88.0
	From 26 to 45 years old	11.0
	Over 46 years old	1.0
Medical history	No	96.0
	Hypertension	1.0
	Depressive status	3.0

*graduated students: student who finished their studies and were waiting to work. ^**^last year student: students who were in the last year of their education for being assistant physicians or nurses. These study subjects (FHWs) had to stop online study (last year student) and work under supervision as nurses or assistant doctors in the field-hospital similar with local official nurses or assistant physicians.

### Self-declared sleep disorders and sleep characteristics of study participants

Most participants claimed insufficient sleep during the working period at COVID-19 field hospitals (64 vs. 26% before). Only 19% of them claimed insufficient sleep during the isolation period. Similarly, there were significant differences between the sleep-time within the 3 periods. The mean sleep-time of FHW before frontline work, during frontline work, and during the isolation period after working at COVID-19 field hospitals was 7.78 ± 1.48, 5.71 ± 1.40, and 8.78 ± 2.31 h per day, respectively.

**Figure 1:**
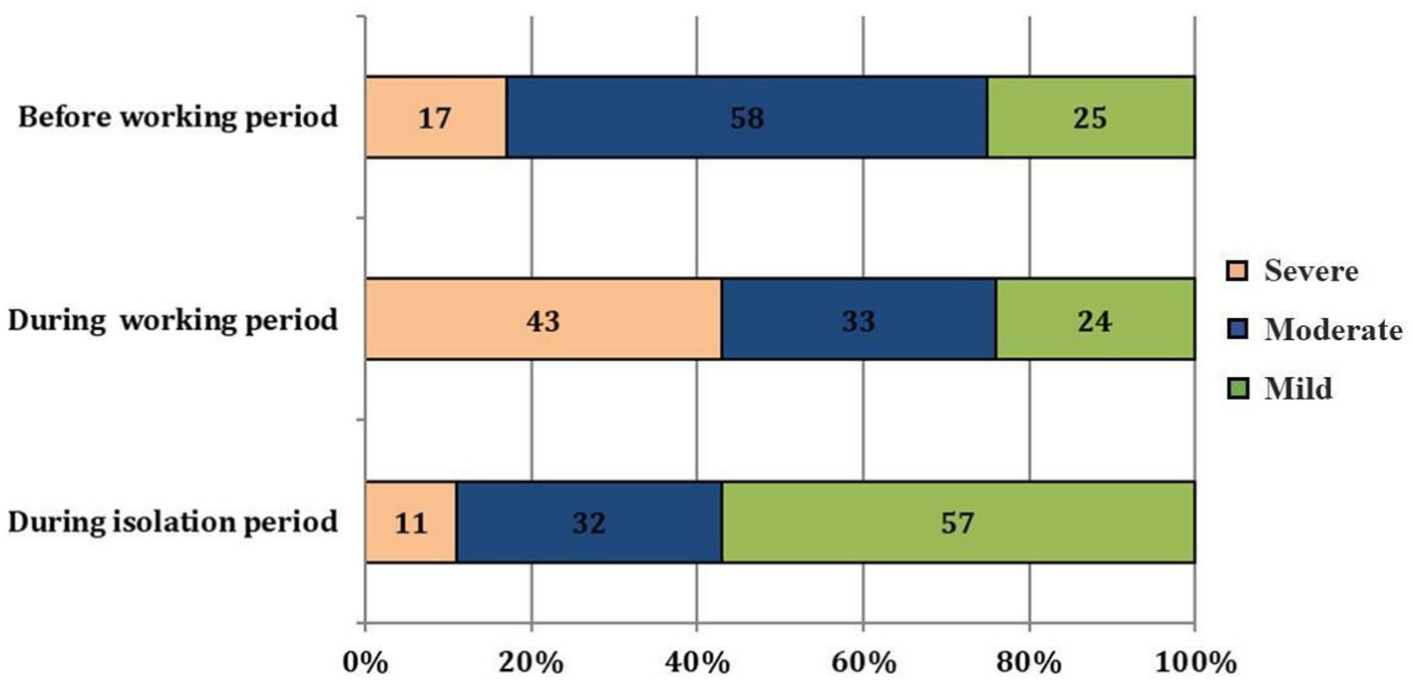
Self-declared sleep disorder severity of study participants.

The difference between before and during the working period was statistically significant with *p* < 0.001. The daily sleep-time during the isolation period was not only longer than during the working period but also the period before frontline work began (*p* < 0.01). Similarly, the severity of sleep disorders was also ameliorated during the isolation (Figure 1). To possibly explain sleep difficulty, FHW revealed some environmental factors and working conditions (Table 2). The night shift was the primary factor influencing the FHW's sleep. The quality of their bed took second place. Reportedly, 43% of participants did not have any influencing factors to their sleep quality (Table 2).

**Table 2:** Factors influencing sleep quality of study participants (*n* = 100).

**Factors**	**Before working period ^(a)^** **(%)**	**During working period ^(b)^** **(%)**	**During isolation period ^(c)^** **(%)**	**p^(a)vs.(*c*)^**	**p^(a)vs.(*b*)^**	**p^(b)vs.(*c*)^**
Light and noise	14	7	0	-	0.053	-
Bed quality	3	15	3	0.001	0.002	0.002
Climate	2	8	3	0.149	0.026	0.21
Mosquito	1	6	5	0.166	0.027	1.00
Night shifts	0	21	0	-	-	-
Nothing	80	43	89	< 0.001	< 0.001	< 0.001

### FHWs fatigue and depression in study participants

The mean score of Pichot's Fatigue Scale was 4.18 (SD = 5.42). The maximum point was 19 and no one was considered as burned out according to this scale (>22 points). The mean value of each element scored varied from 0.33 to 0.65. The Pichot's Depression mean score was 2.54 (SD = 3.36). The highest point was 13 and only 13 subjects scored above 7 for fatigue, which indicated depression. The mean value of each element scored varied from 0.08 to 0.35. The two elements with the lowest mean scores were “feeling oneself at an impasse” and “making every effort” (mean scores: 0.08 and 0.09, respectively. The element with the highest score was “feeling sad” (mean score: 0.35).

Pichot's Fatigue mean score was lower in those who declared they slept enough (3.90, SD = 5.05) vs. those that did not (4.96, SD = 5.55), although it was not statistically significant (*p*= 0.39). Similarly, the Pichot's Depression mean scores in these two groups were also not statistically significant with a mean score of 2.31, SD = 3.04 and 3.19, SD = 4.14 (*p* = 0.25), respectively. The mean values for 3 participants with depression history were 10.67 (SD: 4.62; *p* = 0.03) for fatigue and 4.0 (SD: 5.29; *p* = 0.45) for depression scores.

### FHWs fatigue and depression in the relation with sleep disorders

We compared the differences in the Pichot's scores within the sub-groups in the two following tables (Tables 3, 4). The fatigue scores decreased significantly in the group claiming short sleep latency. Most of the remaining score factors increased slightly, except for “snoring” and “observed sleep apnea.” The factor that increased the depression score was anxious feeling (*p* = 0.001). Other significant factors were “short sleep latency,” “observed sleep apnea,” “tiredness, daily sleepiness” and “snoring”.

**Table 3:** Sleep characteristics and Pichot's Fatigue score of study participants (*n* = 100).

	**Factors**		***n***	**Mean**	**SD**	**t**	***p***
STOP items	Snoring	Yes	11	5.36	4.67	−0.77	0.02
		No	89	4.03	5.51		
	Tired / Daily sleepiness	Yes	44	3.73	5.39	0.74	0.46
		No	56	4.53	5.47		
	Observed (Sleep apnea)	Yes	19	6.74	6.05	−2.33	0.02
		No	81	3.58	5.12		
	Pressure (Hypertension)	Yes	1	8.00	-	-	-
STOP score (+)		Yes	6	5.67	5.24	−0.69	0.49
		No	94	4.08	5.44		
Other sleep characteristics	Fast to sleep	Yes	47	3.00	4.33	2.08	0.02
		No	53	5.23	6.08		
	Fragmented sleep	Yes	31	4.71	5.77	−0.65	0.51
		No	69	3.94	5.28		
	Morning headache	Yes	28	4.82	5.93	−0.74	0.46
		No	72	3.93	5.23		
	Cognitive impairment	Yes	39	4.28	4.79	−0.15	0.88
		No	61	4.11	5.82		
	Anxiety	Yes	30	4.53	5.30	−0.42	0.67
		No	70	4.03	5.50		
	Overweight	Yes	4	3.25	6.50	0.35	0.73
		No	96	4.22	5.40		

**Table 4:** Sleep characteristics and Pichot's Depression score of study participants (*n* = 100).

	**Factors**		**n**	**Mean**	**SD**	**t**	***p***
STOP items	Snoring	Yes	11	4.27	4.43	−1.8	0.07
		No	89	2.32	3.17		
	Tired / Daily sleepiness	Yes	44	3.29	3.62	−2.02	0.04
		No	56	1.95	3.04		
	Observed (Sleep apnea)	Yes	19	4.16	4.40	−2.39	0.02
		No	81	2.16	2.97		
	Pressure (Hypertension)	Yes	1	0.00	-	-	-
STOP SCORE (+)		Yes	6	3.33	4.08	−0.59	0.55
		No	94	2.49	3.33		
Other sleep characteristics	Fast to sleep	Yes	47	1.59	2.13	2.73	0.01
		No	53	3.38	3.99		
	Fragmented sleep	Yes	31	2.74	3.29	−0.40	0.69
		No	69	2.45	3.41		
	Morning headache	Yes	28	3.43	3.83	−1.66	0.10
		No	72	2.19	3.12		
	Cognitive impairment	Yes	39	3.41	3.51	−2.10	0.04
		No	61	1.98	3.16		
	Anxiety	Yes	30	4.20	4.15	−3.40	0.001
		No	70	1.83	2.69		
	Overweight	Yes	4	3.25	5.25	−0.43	0.67
		No	96	2.51	3.29		

## Discussion

The COVID-19 pandemic has put enormous pressure on the healthcare system globally, resulting in the establishment of many field hospitals and isolation camps throughout the world. This temporary solution, with no doubt, has led to the suspension of adequate environmental conditions for patients and for healthcare workers (11), subsequently increasing their risk for infection. Hence, in many articles, FHWs suffered from sleep disorders and mental health disabilities. In a study with renal healthcare practitioners during the COVID-19 social lockdown in Belfast, UK, 35.9 % participants developed severe levels of emotional exhaustion, 16.7% had severe levels of depersonalization, and 21.1% experienced low levels of personal accomplishment (12). A Mexican cross-sectional study reported that of the 507 interviewed healthcare workers, 70.02% were at risk of burnout (13). Furthermore, 57.31, 7.91, and 2.77% had a mild, moderate, and severe risk of post-traumatic stress disorder, respectively. Of the predominantly female population examined in this study, the most commonly affected individuals were female healthcare workers and those diagnosed with COVID-19 or exposed to a person infected with COVID-19 (13).

Surprisingly, no one in our study was classified as exhausted prior to the working period, during the working period, or during the isolation period according to the Pichot's Fatigue score. Our mean score was far lower than the cut-point of the scale (4.18, SD = 5.42 vs. 22). The majority of participants included in this study were identified as female students who were relatively young and in good health. Previous studies demonstrated that the prevalence of sleep problems increased among adolescents and among university students when compared with the general population (14). Furthermore, a study among FHWs in Saudi Arabia showed those with fewer years of experience had higher burnout symptoms (15). Based on this information, young age may not be concluded as the sole protective factor for healthcare workers and work conditions may serve as a more likely explanation. The COVID-19 outbreak happened in Vietnam later than most countries around the world, thus giving the country more time and experience to organize the field work in preparation for the virus. These findings serve as our hypotheses, but the lack of preparation and infrastructure to protect the public and healthcare practitioners might exert pressure on people and the healthcare system (16).

Within the items of Pichot's Depression scores, few participants admitted to “feeling oneself at an impasse” or “feeling drained.” The most common complaint from participants was “sadness.” Indeed, 13 cases were suspected of depression, including those from 3 participants that had previously been diagnosed with depression. In a cross-sectional, self-administered registry enrollment survey performed on US healthcare workers, 41% responded that they were experiencing burnout. In a study from 2021, participants were instructed to respond to the questions on the survey based on the day before they were currently completing it. The results from the survey identified that 53% of participants reported feeling tired over most of the day, 51% felt feelings of stress, 41% had trouble sleeping, 38% experienced worry, 21% experienced sadness, 19% reported physical pain, and 15% felt feelings of anger (17). Another study on pharmacists revealed that 40% experienced more anxiety and 25% experienced more sadness and/or depression during the COVID-19 pandemic (18).

There was no significant difference between the mean scores of clinical suspected cases of obstructive sleep apnea (OSA) and the rest of the cases. However, while analyzing each STOP item, the Pichot's fatigue score was significantly significant for two symptoms of obstructive sleep apnea — “snoring” and “observed sleep apnea.” These two symptoms also slightly increased the depression score. The STOP questionnaire that we used to classify the patients as high or low risk of having OSA was demonstrated to have a high combination of both sensitivity and specificity. Using the apnea-hypopnea index (AHI) with a score >5 as a cutoff value to evaluate it, the sensitivity was 65.6%, the specificity was 60.0%, the PPV (positive predictive value) was 78.4%, and the NPV (negative predictive value) was 44.0% (19). Hence, we suspect that the relationship between Pichot's scores and the STOP items were more so related to the participant's personal state rather than work conditions.

Another related factor to Pichot's scores was “fast to sleep.” Short sleep-onset latency could be a symptom of tiredness, depression, or other sleep disorders. But falling asleep quickly at night is also necessary for health recovery (20). That is why “fast to sleep” appeared as a protective factor against tiredness and depression in our study. Feelings of anxiety and difficulty to recall memories were two of depression's symptoms. These two were also significantly related to Pichot's Depression score in our study, which confirmed our finding. The 13% supposed cases were relatively lower than other studies. Surveys on frontline health workers in China showed that depression symptoms accounted for 34.4% to 50.4 % participants (8, 21, 22). Another study of Rossi et al. (23) in Italy reported a lower rate with 24.7% (23). The result of another survey on 173 healthcare workers at 2 hospitals in Hanoi, Vietnam was also lower than overseas with the rate of depression symptoms being 20.2% (24).

There was also an increase in sleep difficulty and its severity during the working period at COVID-19 field hospitals. About two-thirds of subjects claimed insufficient sleep. The mean sleep-time decreases more than 2 h in comparison with their habitude. As a result, 44% of our participants suffered from tiredness or daily sleepiness due to the FHWs' work demand, especially those with shift work or night shifts. As already established, shift work and night shifts are very common in healthcare organizations worldwide. Here, healthcare professionals doing shift work and night shifts are exposed to several stressors with psychological, social, physical, and sleeping consequences (25). Fortunately, these intensive work conditions didn't relate to our health workers' fatigue as our participants worked only in the field hospital for a short determinate period, however, we understand the importance of recognizing the burden shift work and night shifts place on the permanent practitioners in the hospitals.

The sleep conditions at field-hospitals are also important. Appropriate preparations to help FHWs feel safe at work whilst sleeping are not enough. Instead, basic sleeping conditions required for a good sleep such as the quality of the bed, light, and noise must be improved. It was proved that the evening light environment in hospitals can be designed to produce less disruptive effects on the circadian system and improve sleep (26). Fatigue and daily somnolence are frequently viewed because of non-restorative sleep (27). To prevent burnout, it is imperative to decrease the charge, ameliorate stress levels and to find strategies to promote personal recharge, especially restorative sleep. In our study, the two isolation weeks after working at field-hospitals take place during the restorative period. Our participants slept more and with a higher quality of sleep, than during and even before their work period. Even extending sleep duration does not always clearly correspond to reductions in daytime fatigue or improvements in mood (28). Nevertheless, providing healthcare workers with better control over their work schedules and opportunities for improved sleep may improve their job attitudes (29).

Finally, the present study has several limitations such as the absence of the control group and the skewness in the data due to the sample consisting of almost 90% female subjects and 94% of study subjects were last year students and graduated students from medical college. Therefore, the results of this study did not represent the spectrum of healthcare workers in Vietnam during COVID-19 pandemic. In addition, the mobilization of medical students, who did not have enough experience to participate in the care of patients contracting COVID-19, during the epidemic as assistants or working under supervision may not be as common elsewhere. The small sample size was also an additional limitation of the present study because it limits the analysis of the characteristic differences between sub-groups. Hence, similar studies with large and representative samples are necessary in the future to demonstrate the mental and sleep disorders of FHWs during a pandemic.

## Conclusion

The majority of young volunteer healthcare workers have passed through the working period at COVID-19 field-hospitals with exhaustion and/or depressive mood. This problem might be due to poor quality sleep, sleep disorders' influence, or fatigue. Pre-established conditions such as appropriate work schedule, better sleep conditions, and mental health support for FHWs during the COVID-19 pandemic are crucial. Thus, the mandatory 2 weeks of isolation after working in field hospitals may provide the opportunity for FHWs' recovery.

## Data availability statement

The raw data supporting the conclusions of this article will be made available by the authors, without undue reservation.

## Ethics statement

The studies involving human participants were reviewed and approved by Institutional Review Board (IRB) of Vietnam Society of Sleep Medicine (VSSM-03.2021). The patients/participants provided their written informed consent to participate in this study.

## Author contributions

SD-Q, ST-D, KB-D, DH-C-B, QV-T-T, and VN-N: conceptualization, validation, and writing—original draft preparation. SD-Q, ST-D, and VN-N: methodology and writing—review and editing. SD-Q, ST-D, and QV-T-T: Software. SD-Q, ST-D, KB-D, and DH-C-B: formal analysis. All authors contributed to the article and approved the submitted version.

## Conflict of interest

The authors declare that the research was conducted in the absence of any commercial or financial relationships that could be construed as a potential conflict of interest.

## Publisher's note

All claims expressed in this article are solely those of the authors and do not necessarily represent those of their affiliated organizations, or those of the publisher, the editors and the reviewers. Any product that may be evaluated in this article, or claim that may be made by its manufacturer, is not guaranteed or endorsed by the publisher.
